# Drug and Therapeutics Committee (DTC) evolvement and expanded scope in Ethiopia

**DOI:** 10.12688/gatesopenres.13200.2

**Published:** 2023-03-10

**Authors:** Habtamu Seyoum, Zinabie Feleke, Dinkineh Bikila, Alebel Yaregal, Amsalu Demisie, Seid Ali, Salem Fisseha, Yigeremu Abebe, Audrey Battu, Felix Lam, Regasa Bayisa

**Affiliations:** 1 Child Survival Program, The Clinton Health Access Initiative- Ethiopia, Addis Ababa, Ethiopia; 2 SRMNCH - Global Essential Medicines, The Clinton Health Access Initiative-Global, Boston, USA; 3 Pharmaceutical and medical equipment directorate, Ministry of Health – Federal Democratic Republic of Ethiopia, Addis Ababa, Ethiopia

**Keywords:** DTC, Establishment, Functionality

## Abstract

As a key partner of Ministry of Health (MOH) Ethiopia, The Clinton Health Access Initiative (CHAI) had been implementing the Child Survival Project (CSP) since October 2015. Strengthening DTC was one of its focuses to improve overall supply chain management (SCM). The objective of this study are to review the evolution of DTCs in Ethiopia from their early years to current practice and identify the major driving and hindering factors for their functionality.

A descriptive mixed study design was employed. The study made use of qualitative data supplemented with quantitative data, generated from both primary and secondary sources through key informant interviews and desk review methods.

DTCs were introduced in Ethiopia in the early 1980s. The mandate of DTCs has been given to four different government organizations during that time. As a result, due to a lack of coordination among these organizations, its implementation was lagging. Recently, the government and its partners have given attention to DTCs. More than 5847 professionals underwent DTC training from 2016 onwards. DTC establishment in health facilities improved from 85% to 98% between 2015 and 2019 during baseline and end-line assessments carried out by CHAI/CSP. Similarly, DTC functionality in HFs improved from 20% to 63%. The CHAI/CSP regular supervision data analysis revealed that DTC establishment improved from 83% to 100% of HFs, while its functionality improved from 5% to 72% between 2016 and 2019, respectively. A chi-square test of independence examining the relationship between facility and pharmacy head training on DTCs and functionality of DTC in the same facility revealed a significant association between the two variables at p<0.0001.

**Conclusions:** Providing consistent capacity building and availing strong monitoring and evaluation system improves functionality of DTCs. Moreover, national coordinating bodies for DTCs and similar structures at Regional Health Bureaus and woreda health offices should be established.

## Abbreviations

BMGF, Bill & Melinda Gates Foundation; CHAI-E, Clinton Health Access Initiative- Ethiopia; CSP, Child Survival Program; DACA, Drug Administration and Control Authority of Ethiopia; DTC, Drug and Therapeutics Committee; EFDA, Ethiopian Food and Drug Administration; EHCRIG, Ethiopian Health Center Reform Implementation Guidelines; EHRIG, Ethiopian Hospital Reform Implementation Guidelines; EPSA, Ethiopian Pharmaceutical Supply Agency; FMHACA, Food, Medicine and Health Care Administration and Control Authority; HF, health facility; HSDP, Health Sector Development Program; HSTP, Health Sector Transformation Plan; IP, implementing partner; IPLS, Integrated Pharmaceuticals and Logistics Systems; KII, key informant interviews; M&E, monitoring and evaluation; MOH, Ministry of Health; MSH, Management Science for Health; PTC, Pharmacy and Therapeutics Committee; PFSA, Pharmaceutical Fund and Supply Agency; RDU, rational drug use; RHB, Regional Health Bureaus; RRF, reporting and requesting form; SCM, supply chain management; SNNPR, Southern Nations Nationalities People’s Region; SOP, standard operating procedures; SS, supportive supervision; TOT, training of trainers ; TWG, technical working group; USAID, United States Agency for International Development; WHO, World Health Organization; WoHO, Woreda health office; ZHD, zonal health department.

## Introduction

### Background

A Drug and Therapeutics Committee (DTC) is a platform for organizing multi-disciplinary professionals in a given health facility (mainly hospitals and health centers (HCs)) to improve the sustainable availability and rational use of essential medicines and medical devices at health facilities (HFs) ^1^. According to the World Health Organization (WHO), the goal of DTC is to ensure that patients are provided with the best possible cost-effective and quality care through determining what medicines will be available, at what cost, and how they will be used ^2^. A DTC is an important tool for improving healthcare delivery.

Pharmacy and Therapeutics (P&T) committees came into existence almost a century ago as a forum for discussing drug use in hospitals in high-income countries including Australia, the USA and European countries. In Australia, 92% and in the UK (in 1990), 86% of hospitals had developed some type of hospital therapeutic committee ^3^. In the USA, DTCs or similar committees are required in the management of the formulary and the authorization and restriction of new drugs ^4^. Following the irrational use of medications in hospitals in low- and middle-income countries, developing DTCs for hospitals was suggested as a starting point to act as an agent of change. This recommendation was forwarded from the first International Conference on Improving Use of Medicines (ICIUM), which was conducted in Thailand in 1997, taking lessons from high-income countries, where more has been documented about the effectiveness of such committees ^5^. Since 2001, the WHO, in collaboration with Management Science for Health, Rational Pharmaceutical Management (RPM) Plus, developed the first Drug and Therapeutics Committees (DTC) Training Course, which was revised in 2007, and then the first DTC practical guide was issued by WHO in 2003 ^2, 6^. Currently, huge numbers of health facilities (HFs) both in developing and developed countries have established DTCs.

Ethiopia started establishing and strengthening DTCs in the 1980s. The first DTC guideline was issued by the MOH in 1986 and this was revised by the Drug Administration and Control Authority of Ethiopia (DACA) in 2004 ^7^.

DTC is an important tool for improving healthcare delivery. Furthermore, it is risky to concentrate too much power over the pharmaceutical system in a single body, as this may lead to corrupt practices. The functions of selecting medicines, procurement, payments and inventory control are best kept separate. Hence, the DTC is an essential component of a HF for medicine selection, use, and distribution of activities and rational use follow-up ^2^.

### Efforts, achievements and gaps in the establishment and strengthening of DTCs in Ethiopia

It is a well-known fact that a DTC is an ideal and cross-cutting health systems strengthening tool in a given HF or country because of its broad mandates in pharmaceutical supply chain management (SCM) and rational drug use (RDU). With this understanding, the MOH Ethiopia, in collaboration with Regional Health Bureau (RHB) structures, its agencies and implementing partners (IPs), invested a lot in the establishment and strengthening of DTCs in Ethiopia. A DTC guideline was developed and revised at different times. DTCs were mentioned in national strategic documents like the Health Sector Development Program (HSDP) II, HSDP III, HSDP IV and Health Sector Transformation Plan (HSTP). It is also widely addressed in Ethiopian Hospital Reform Implementation Guideline (EHRIG) and HC Reform Implementation Guidelines (EHCRIG) with requirements that each HF establish a DTC to promote the safe, rational and cost-effective use of medicines. Recently, the DTC guidelines and standard operating procedures (SOPs) were revised and tailored to a user-friendly and action-oriented approach. Using the revised SOP, the MOH and the Ethiopian Pharmaceuticals Supply Agency (EPSA) in collaboration with IPs have organized a number of cascading training sessions including training of trainers (TOT) for health professionals (physicians, pharmacists, health officers and nurses) on DTCs.

A survey conducted in 2013 on the Functional Status and Perceived Effectiveness of DTCs at Public Hospitals in Ethiopia revealed that more than 90% of public hospitals established a DTC ^1^. In addition to that, partners’ supportive supervision (SS) and survey reports revealed similar findings in the establishment of DTCs at HFs (hospitals and HCs). A recent DTC national assessment conducted at the end of 2018 revealed that all health centers 40(100%) and 25(96.2%) hospitals established DTC at the time of the assessment. Moreover, DTC members were assigned by official letter in 23(88.5%) hospitals and 32(80%) health centers ^8^. However, the majority of the so-called established DTCs are not functional. For example, a study conducted in 2015 by the MOH and CHAI Ethiopia revealed that less than 20% of HFs have functional DTCs, with 25% of hospitals without functional DTCs ^9^. In that assessment, the operational definition of a functional DTC was if it had terms of reference (TOR), conducted regular DTC meetings (meet at least every two months) and had documented DTC meeting minutes. Similarly, training of both the pharmacy head and facility head, which is the standard for DTC training, is low (below 20%). The MOH study revealed that only about 20 (18.5%) of the hospitals with DTCs had both a chairperson and secretary trained on DTCs in 2013 ^1^ and the study conducted by CHAI after two years in 2015 also showed that only 7% and 13% of HFs and only 17% and 33% of hospitals have a facility head and pharmacy head trained on DTCs, respectively ^1, 9^, though it was assumed a large number of professionals had been trained on DTCs since 2006 by the MOH and partners.

### Rationale for the study

As one of the key partners of the MOH in Ethiopia, CHAI has been implementing the Child Survival Program (CSP) since October 2015 in collaboration with the MOH and EPSA in four agrarian regions of Ethiopia, namely Amhara, Oromia, SNNPR and Tigray regions. Although primarily focused on diarrhea and pneumonia, CSP interventions were designed to support broader supply chain issues, including the improvement of Integrated Pharmaceuticals Logistics Systems (IPLS), DTCs and supply chain functions including quantification, procurement, distribution and rational use of essential medicines. Particularly, CHAI has put significant effort into supporting the revitalization of DTCs. CHAI supported the establishment and/or strengthening of DTCs in selected HFs (about 2000 HFs (N=1600 HCs) and (N=400 hospitals)) in 400 intervention woredas (districts).

CHAI, in collaboration with the MOH, set out to (1) review the DTCs in Ethiopia and (2) generate evidence to guide future investment. Hence, it was worth conducting DTC-specific studies and forward viable recommendations that could be an input for national policy and strategies on DTCs.

## Objectives of the study

### General objective

The general objectives of this study are to review the evolution of DTCs in Ethiopia from their early years to current practice; identify the major driving and hindering factors for their functionality and impact on the pharmaceutical SCM system and pharmacy service and propose feasible recommendations.

### Specific objectives

The specific objectives of this study are to:

1. Review the history of DTCs in Ethiopia
2. Identify the major driving factors/process for DTC impact on health care improvement in Ethiopia
3. Identify the barriers for DTC functionality in HFs in Ethiopia
4. Forward recommendations to strengthen DTCs across the country

## Methods

### Scope of the study and study period

A desk review of available national and international documents between the introduction of DTCs in the 1980s into the country to 2019 was conducted. Documents reviewed for this study included government DTC related guidelines, SOPs, WHO documents, partners’ monitoring and evaluation findings, SS and survey reports and training databases. Additional data was gathered from key informant interviews (KIIs) with senior officials and professionals from EPSA, MOH, RHBs and IPs. The study was conducted from May 20, 2019 to June 20, 2019.

### Study design

A descriptive mixed method study design that employed both qualitative and quantitative data collection methods was used.

### Data type and sources

The assessment made use of both qualitative and quantitative data, generated from primary and secondary sources and collected through KIIs and desk review methods, respectively.

### Sampling technique

A purposive sampling method was applied to select the study participants/informants and institutions as well as to select documents for review. The selection of interviewees was made in consultation with relevant government office representatives for the in-depth KIIs.

### Data collection tools

Semi-structured interview guides were used for the KIIs with open-ended questions to shape the discussion. The semi-structured interview guides were pretested with individuals with similar educational and work experience background. Their feedback was incorporated into the interview guide.

### Data collection technique

Combinations of primary and secondary data were collected using various data collection techniques described in detail below.

### Desk reviews

Conducting a desk review was one of the core components of the assessment task. By collecting, organizing and synthesizing available information, the team gained an understanding of DTC contexts and trends, and equally importantly, identified gaps to forward viable recommendations. The assessment team reviewed available relevant national and other documents from MOH, RHBs, EPSA, CHAI, WHO and other relevant partners as supporting evidence of the assessment. These documents were identified from purposively selected relevant organizations that have been working on DTCs and SCM activities. In addition, the selected documents from those organizations were specific to DTC implementation. Mainly, secondary data were reviewed as the quantitative data component of the assessment. The quantitative component of the assessment was conducted to triangulate and strengthen the qualitative findings. In this review, training databases from MOH and CHAI were reviewed. Moreover, data and reports from baseline and end-line assessments conducted by CHAI in Ethiopia were reviewed. Joint EPSA/RHBs and CHAI SS reports and databases were also reviewed (see all the three databases as *Underlying data* ^10^). Other documents reviewed for this study were DTC guidelines, manuals, SOPs, training materials and related survey reports.

### Key informant interview

This qualitative study involved the collaborative efforts of a multidisciplinary research team of medical doctors, internists, senior pharmacy professionals and public health professionals with extensive experience in project evaluations and operational research. On average, the team members have 17 years of experience, ranging from 10 to 38 years, working mainly in the MOH structures with different responsibilities. Moreover, the KIIs were conducted by senior pharmacists, who have extensive experience in pharmaceutical SCM and pharmacy services including DTCs, coupled with ample experience in both qualitative and quantitative data collection. Such a mix of Monitoring and Evaluation (M&E) and pharmacy professionals in the research team gave the opportunity to ask pertinent questions to the KII participants and gain deep insight into the study. Furthermore, the KII participants were selected purposively based on their current and/or previous position in relation to DTCs. Appropriate experience of the research team contributed a lot to identifying suitable KII participants as well as to a smooth relationship with participants and to the virtue of the study. Additionally, the KII participants contacted to take part in the study, in addition to the role and position held, were knowledgeable and had an objective eye for the organizations they represent. Eligible respondents for the KIIs were initially identified from EPSA, MOH, RHBs and IPs. The selected individuals were contacted to explain the purpose of the study.

### Data collection process and setting

Prior to the actual interview date, participants were invited with an official letter and telephone call to arrange appointments. Based on their appointment date and time, the research team/data collectors reached at the participants’ office. Almost all the KII interviewees were high-level managers and senior officials. Hence, they had their own private offices in their respective institutions. The interviews were conducted face-to-face in their offices privately at their convenience. However, if there were two experts in the same office who were to be interviewed for the DTC study, both of them were interviewed as a group in that office. On average, each interview took two hours, ranging from 1.5–2.5 hours. When the data reached saturation, the data collectors heard the same response again and again, interviewing of more participants was quitted. Until that 21 informants were interviewed.

### Data analysis methods

The audio-recorded interviews and discussions were transcribed/translated from the languages of the interviewees into English for analysis. Then, a summary of each KII was developed and organized. The qualitative data analysis involved thematic categorization of transcribed and translated in-depth interviews. Data were analyzed and compiled using a thematic approach (based on the different components of the KII) by conducting content analysis. Finally, narrative analysis was applied to merge most related segments of the findings that were summarized thematically. For quantitative components of the secondary data, relevant, clean and finalized data were accessed from those organizations’ data store. Statistical Package for Social Sciences (SPSS) version 24 was applied for descriptive data analysis and some statistical tests like the Chi-Square test of independence was used to determine if there is an association between categorical variables.

To ensure data security and confidentiality, both the qualitative and quantitative data were de-identified which ensure the anonymity of KII participants and protect personal information from the secondary data. After data analysis activities were carried out, all data types including field notes, audio recordings and data in soft copy formats were archived and stored in secured places with access limited to authorized persons.

### Ethics statement

The DTC evolution study intended to assess system level information and would not reveal sensitive information about individuals or organizations. However, since the findings of this study will be published in international journals, ethical clearance from Ethiopian Public Health Institute (EPHI) was requested and granted with the ethical approval number EPHI-IRB-175-2019, dated June 24, 2019. EPHI is the national Institutional Review Board (IRB) through its Scientific and Ethical Review Office (SERO), in charge of ethical review and approval of health and nutrition related research in Ethiopia. Moreover, data collectors/interviewers were trained in ethical data collection and confidentiality. Each participant was asked and agreed to participate voluntarily in the interview through written informed consent.

### Dissemination of findings

The final DTC evolution study report was shared with relevant government (MOH, RHBs key agencies) bodies and IPs. A summary of the report also will be presented at different events like relevant workshops and regular review meetings. In parallel, international journals will be identified and the abstract will be sent for publication.

## Results

### Interview participants’ backgrounds

A total of 22 KII participants were contacted and interviewed. The participants were selected based on their ample experience, exposure and their current position in relation to DTCs. On average, the KII participants have more than 12 years’ of experience in DTC related implementation. During the time of the study, these KII participants are working at MOH (1); EPSA central office (1); RHBs (6); EPSA regional hubs (3; Bahir Dar, Mekelle and Hawassa); EFDA (1) and partners working/supporting or experienced in DTC implementation (9), namely the private sector, WHO, United Nations Population Fund, Promoting the Quality of Medicines Plus (PQM+) Program, Global Public Health/ United States Pharmacopeia, United States Agency for International Development (USAID)/Chemonics/PSM, USAID Transform Health in Developing Regions, USAID/AIDS/Free and United States Pharmacopeia.

Apart from their current position, the majority of participants have worked at middle and higher levels of government structures (HF, zonal health department (ZHD), RHB and EPSA hubs). The majority of participants also revealed that they have participated in the development of DTC guidelines, training materials and delivering DTC training for professionals. Many have completed training of trainers (TOT) courses on DTC. Some of them had participated in policy-level support such as Standard Treatment Guideline (STG) development and were part of the team involved in the development of the pharmacy chapter of the Ethiopian Hospital Reform Implementation Guidelines (EHRIG) and Ethiopian Health Center Reform Implementation Guidelines (EHCRIG).

### History of DTCs in Ethiopia

KII participants replied that DTCs are one of the oldest multidisciplinary platforms in Ethiopia HFs, next to HF management committees. They were introduced to Ethiopia in the early 1980s as Pharmacy and Therapeutics Committees (PTCs). At that time PTCs/DTCs served as a tool for hospitals to facilitate routine activities such as disposal and procurement of medicine and addressed some quality issues but lacked suitable implementation guidelines and operational definitions. Later on, in 2006, DTCs were shaped with training of professionals and incorporated specific components of rational drug use and pharmacy services like adverse drug reactions (ADR), antimicrobial resistance (AMR) containment, formulary list preparation, drug information system (DIS) establishment, Drug Use Evaluation (DUE), ABC/VEN Analysis for budget allocation and promotion of essential health commodity availability. The training was conducted by DACA/FMHACA/EFDA in collaboration with a partner, Rational Pharmaceutical Management Plus (RPM Plus).

**Figure 1.**
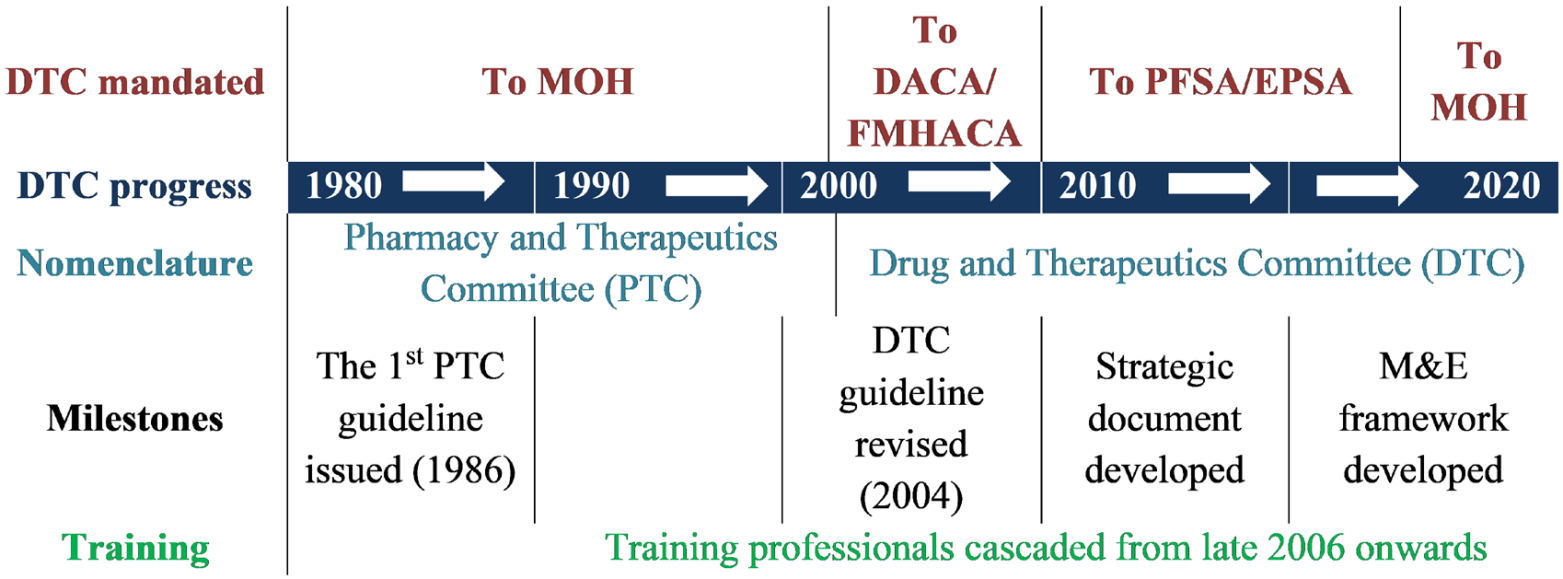
Evolution (milestones) of DTCs in Ethiopia. DTC, Drug and Therapeutics Committee; MOH, Ministry of Health; DACA, Drug Administration and Control Authority of Ethiopia; FMHACA, Food, Medicine and Health Care Administration and Control Authority; PFSA, Pharmaceutical Fund and Supply Agency; EPSA, Ethiopian Pharmaceutical Supply Agency; PTC, Pharmacy and Therapeutics Committee; M&E, monitoring and evaluation.

In the beginning, the mandate of PTCs was given to the MOH and then in 2004 it was given to Drug Administration and Control Authority of Ethiopia (DACA)/FMHACA, the current EFDA. In 2010, the mandate of DTC administration was shifted from FMHACA/EFDA to EPSA (formerly called Pharmaceutical Fund and Supply Agency, PFSA). From late 2017 onwards, the mandate of DTC returned to the MOH, as depicted in Figure 1. This frequent change of mandate from one organization to another could have its own impact on the poor implementation of DTCs in the country. Although DTC platform has been in place for more than 30 years in Ethiopia, its implementation is not comparable with its age. The last 10 years have seen a renewed focus on DTCs. The government has worked with implementing partners such as Management Sciences for Health (MSH) and in the last 3–4 years with CHAI to strengthen DTC implementation. Partnering with government partners, particularly EPSA and MOH, has enabled CHAI through its Child Survival Program to support a large number of HFs to establish DTCs.

### DTC related policies and current practices

***Policy and regulation*.** Regarding policy and regulation, participants replied that the first PTC guidelines issued by the MOH in 1986 focused on drug supply management topics and was mainly adapted from WHO guidelines. Later on, it was revised by DACA in 2004, but its implementation was delayed. In 2011, EPSA (PFSA) tried to develop a strategic document for national and regional advisory committees for DTCs with content on awareness, strengthening and an M&E framework. At the end of 2011, a breakthrough discussion/workshop was organized by EPSA (PFSA) with Regional Health Bureaus (RHBs) on DTC strengthening. Even though DTCs and pharmacy services were neglected in the 1 ^st^ hospital *Blueprint,* the 2 ^nd^ version of EHRIG incorporated a pharmacy chapter that comprehensively addressed both the pharmacy services/DTCs and SCM. From 2016 onwards, the MOH in collaboration with its IPs invested a lot in material revision (training guidelines, DTC SOP), checklist development and M&E framework development.

***Partnership and collaboration*.** There are a number of partners who have been supporting DTCs in Ethiopia directly or indirectly. These include but not limited to WHO, MSH, through Strengthening Pharmaceutical Systems (SPS) and Systems for Improved Access to Pharmaceuticals and Services (SIAPS) projects; Essential Medicine/Child Survival Program of CHAI, USAID/JSI Deliver, USAID/AIDS Free, USAID/Chemonics/PSM and USAID/Transform Health in Developing Regions.

***DTC training*.** With regard to the history of DTC training curriculums, KII participants reflected that in 2004 DACA, in consultation with Africare, conducted a baseline assessment in Addis Ababa City Administration. The first training curriculum was developed, and five days training was provided to five professionals (MD, lab, matron, pharmacy & clinical team leaders) from each hospital in Addis Ababa on setting up a Drug Administration Committee to address gaps in HFs. In 2006, a mini assessment of hospitals was conducted; then the national TOT for 21 hospitals was conducted for 21 days using the WHO/MSH DTC implementation guidelines. The training materials were then customized to make the training cost-efficient and to easily cascade trainings. Based on the revision and customization, a five-day training course was developed (three days on DTC + two days on DIS) and provided to 222 professionals in six rounds in different cities around the country. Then, from 2008 to 2011, MSH/SIAPS, in collaboration with other partners, provided DTC training for a total of 1190 health professionals. After 2012, EPSA and MOH, in collaboration with IPs like CHAI and USAID’s Global Health Supply Chain Program Procurement and Supply Management project (USAID/GHSC-PSM), revised the DTC training materials and provided a national TOT on DTCs and basic DTC training to 1073 professionals. As per the CHAI-Ethiopia CSP training database ^11^, CHAI in collaboration with RHBs and EPSA hubs, provided basic DTC training for a total of 5847 professionals from 2016 to 2019, after providing DTC TOT for a total of 79 professionals to strengthen the regional training pool ^11^. Despite the fact that some training data might be missed, from 2007 to 2019 more than 8400 professionals were trained in basic DTC. In spite of this, the availability of trained professionals in the HFs is affected by the high turnover of staff at HFs. The CHAI-Ethiopia CSP baseline versus endline assessment revealed a 38% improvement in the availability of DTC-trained HF heads and pharmacy heads on the day of visit ^9^ and 12, which is the standard for DTC training (see Table 1).

**Table 1.** Availability of Drug and Therapeutics Committee (DTC) trained health facility (HF) heads and pharmacy heads on the day of visit.

Assessment type	Assessment year	% of HFs with both the HF head and pharmacy head trained on DTC		Number of HFs visited
		Yes	No	
**Baseline**	**2015**	5%	95%	314
**Endline**	**2019**	43%	57%	314

***Research/assessments, M&E on DTC*.** There is limited research on DTCs in Ethiopia beyond small scale assessments. The first large scale assessment, entitled the “National Assessment on Functional Status and Perceived Effectiveness of Drug and Therapeutics Committees at Public Hospitals in Ethiopia” was conducted across the country in a total of 111 public hospitals in 2013. This assessment was led by EPSA; however, this study did not include HCs ^1^. Another study conducted by Gebremariam *et al.* entitled “Assessment of Medicine Supply Management and its Quality Assurance Practice in HFs in Southwest Shoa Zone, Oromia Regional State, Ethiopia“, assessed 10 HFs from March 1 to 12, 2018 and reviewed their DTC practice ^13^. The most recent and relevant DTC assessment was conducted by the MOH in 2018, entitled with “Assessment on Status of Drug and Therapeutics Committee in Public HFs of Ethiopia” in 66 public HFs (26 hospitals and 40 HCs) ^8^.

With regard to M&E of DTCs, there were no well-organized M&E tools in the country. Recently, CHAI-Ethiopia/CSP in collaboration with EPSA and RHBs developed a well-structured SS checklist for HF visits.

CHAI-Ethiopia/CSP was providing regular (bi-annual) SS directly to 400 intervention woredas for 400 selected model HFs through the organization’s staff (though led by CHAI staff, it was conducted in collaboration with RHB, EPSA, ZHDs and WoHOs) and indirectly through woreda logistics officers for about 1200 HFs. The presence of woreda logistics officers in the SS team was mandatory, with the main purpose of providing technical support for HFs and in the meantime building capacity of woreda logistics officers, so as to enable them to conduct similar technical support for their respective catchment HFs independently. During joint supportive supervision (JSS), teams collected SCM and DTC performance data for monitoring of program achievement while providing technical support for HFs. A total of six rounds of SSs were conducted from 2016 to 2019 ^14^.

**Figure 2.**
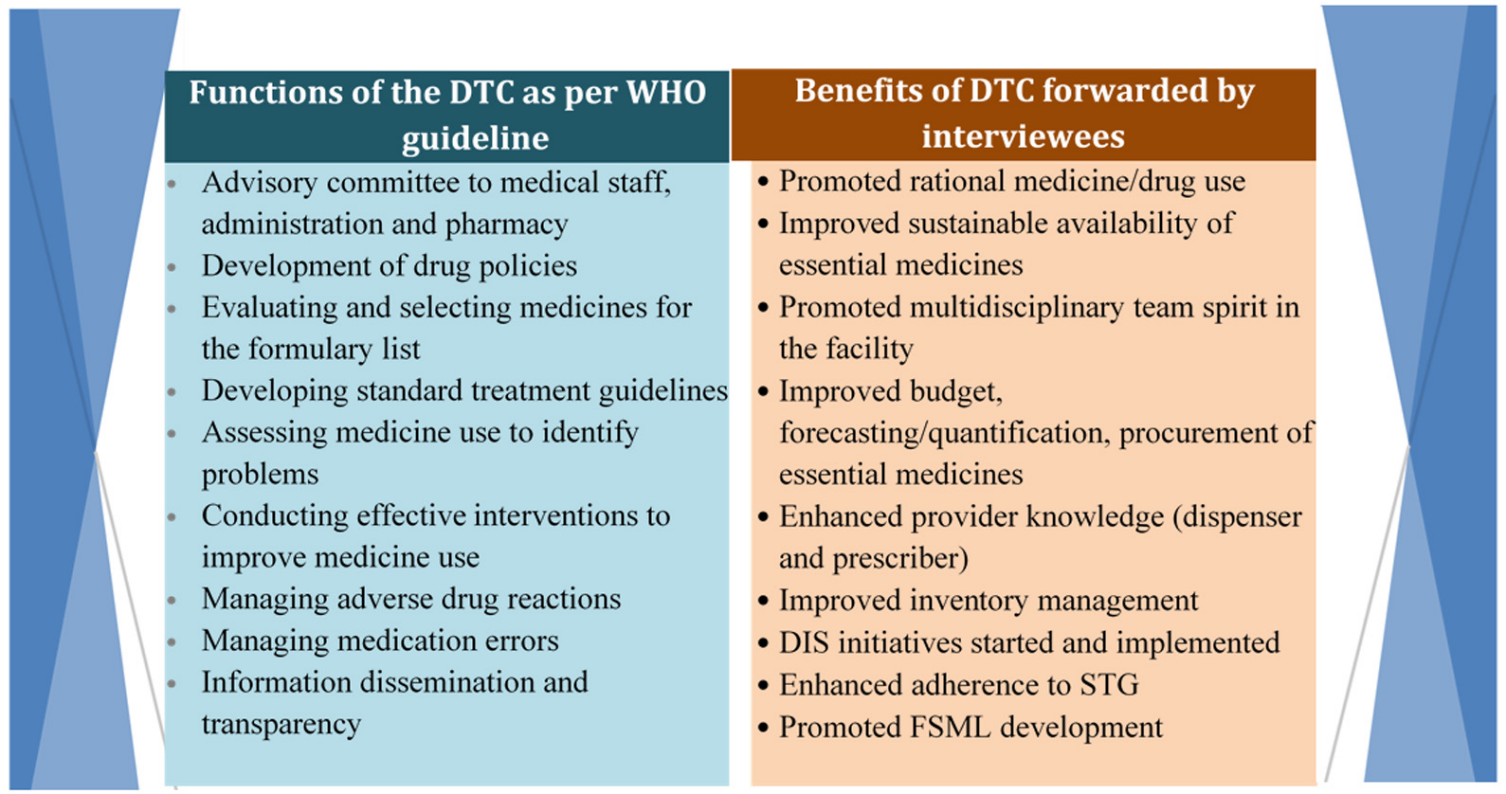
Benefits of DTC as per the interview’s response compared with DTC functions in WHO guidelines. DTC, Drug and Therapeutics Committee; DIS, drug information system; STG, standard treatment guideline; FSML, Financial Services Markup Language; WHO, World Health Organization.

Interviewees also forwarded their testimonies about the major benefits of DTCs. These points are summarized and presented in the table below, compared with the WHO functions of DTC ( Figure 2).

One interviewee from United States Pharmacopeia with about 30 years DTC exposure witnessed *“I assume that almost all hospital and some HFs should have facility level specific medicine list by now, because availability of commodities increased from time to time, prioritization of medicine/resource allocation, RDU, AMR prevention and containment improved as well; some hospitals even developed RDU policy, antibiotics use policy…”*

RHB KII participant stated, *“As the performance of DTC is highly linked with other health care initiatives like APTS, CBHI, Model Pharmacy, RDF, clinical pharmacy, AMR DIC/DIS and so on, without DTC transformation ^1^, there is no health program transformation or no woreda transformation!”*

One partner KII participant also stated *“Good to think as one committee for one HF as DTC is one of the largest committees to address all facility problems”*

### Progress in scaling up DTC in Ethiopia

***DTC establishment and functionality*.** KII participants revealed that in the beginning of 1980s, as the number of hospitals was few (<73), all hospitals had established a PTC. In 2008, out of 112 hospitals, about 83 hospitals had an established DTC. Although the number of HFs increased and DTC training was also cascaded to HFs, until 2012, only 320 HFs were able to establish DTCs. At that time (2012), there were 2,884 public HFs (195 hospitals and 2689 HCs) in the country ^15, 16^. Currently, as per the estimation of the KII respondents, the majority of public HFs, about 90% of hospitals and 87% of HCs, have an established DTC. When this study was conducted (June 20, 2019) there were 4058 public HFs (346 hospitals and 3712 HCs) in Ethiopia that are eligible for DTC implementation ^17^. DTCs, however, have not yet started in private HFs although the DACA/FMHACA/EFDA have tried to push private hospitals to establish DTCs since 2008-2009. Regarding functionality, participants estimated that 54% of HCs and 84% of hospitals had a functional DTC based on the established criteria. The three main criteria for DTC functionality, taken for this study are (1) having DTC TOR, (2) conducting regular (at least every two months) meetings and (3) properly recording and documenting meetings in the facility. The KII participants’ estimation was consistent with the PFSA assessment and the CHAI Ethiopia CSP assessments that DTC establishment and functionality has improved well in public hospitals and HCs. The national survey revealed that 97.8% of public hospitals had established DTC. The majority (87%) of the established DTCs had TOR. The CHAI-Ethiopia/CSP baseline and endline assessment comparison revealed DTC establishment, and particularly functionality, has improved significantly in the last three years in its intervention HFs. Accordingly, DTC establishment increased in public HCs from 85% in 2015 to 98% in 2019 ( Table 2).

**Table 2.** Drug and Therapeutics Committee (DTC) establishment improvement in public health facilities (HFs) in four agrarian regions of Ethiopia.

Assessment type	Assessment year	% of HFs that established DTCs		Number of HFs visited
		Yes	No	
**Baseline**	**2015**	85%	15%	314
**Endline**	**2019**	98%	2%	314

Similarly, the functionality of DTCs for selected indicators (1) availability of TOR, (2) conducting regular meetings to review its performance and (3) documenting the meeting minutes increased significantly, as depicted in Table 3 below.

**Table 3.** Drug and Therapeutics Committee (DTC) functionality improvement in public health facilities (HFs) in four agrarian regions of Ethiopia.

Assessment type	Assessment year	% of HFs that had functional DTCs		Number of HFs visited
		Functional	Not established or not functional	
**Baseline**	**2015**	17%	83%	314
**Endline**	**2019**	62%	38%	314

**Figure 3.**
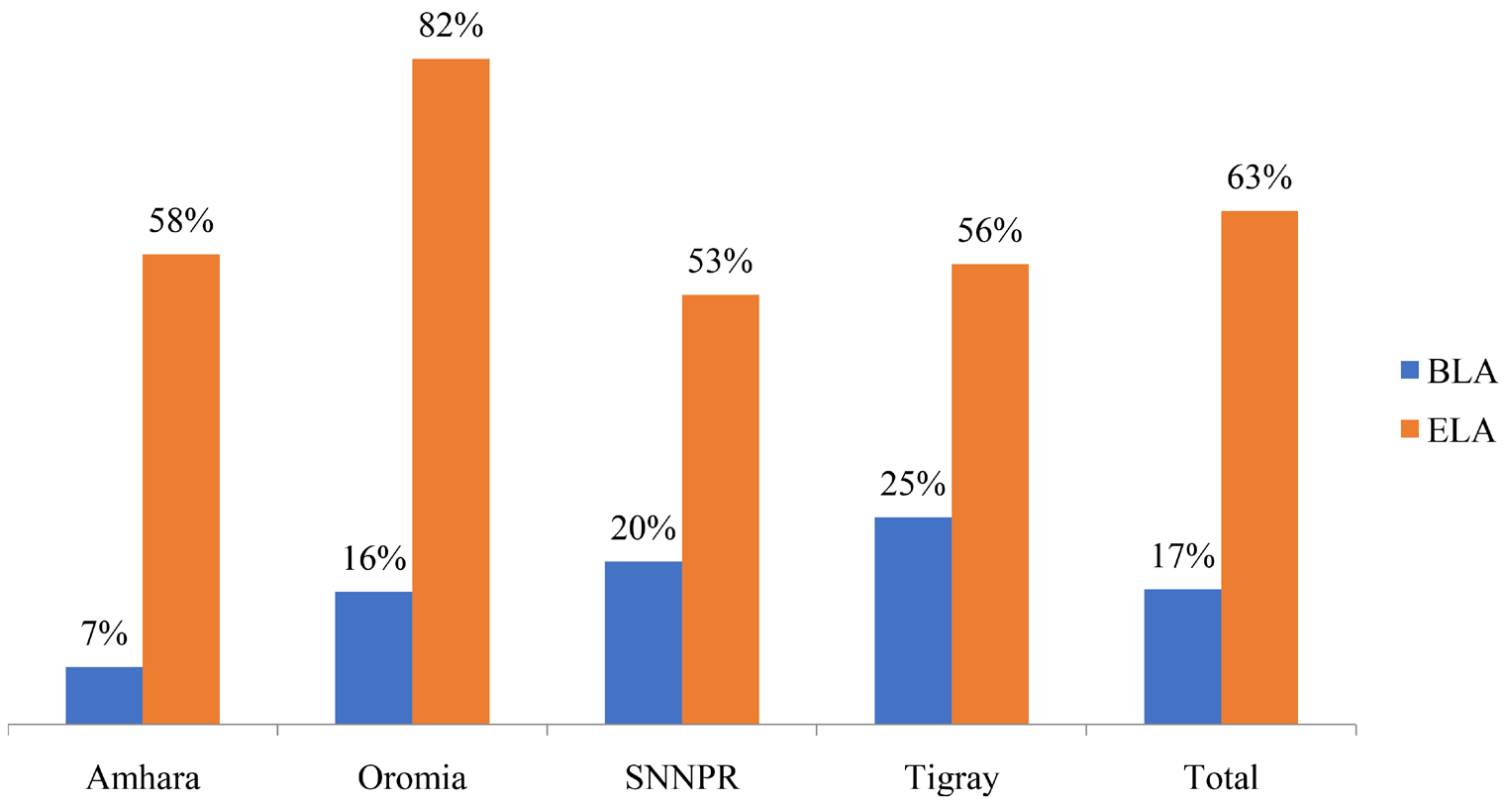
Functionality of DTC BLA Vs ELA by region.

A study conducted by FMOH and EPSA in 2014 revealed that only about 20(18.5%) of the hospitals with DTC had both chairperson and secretary trained on DTC ^1^. Regional performance status and comparison between BLA and ELA is summarized in ( Figure 3) below

**Figure 4.**
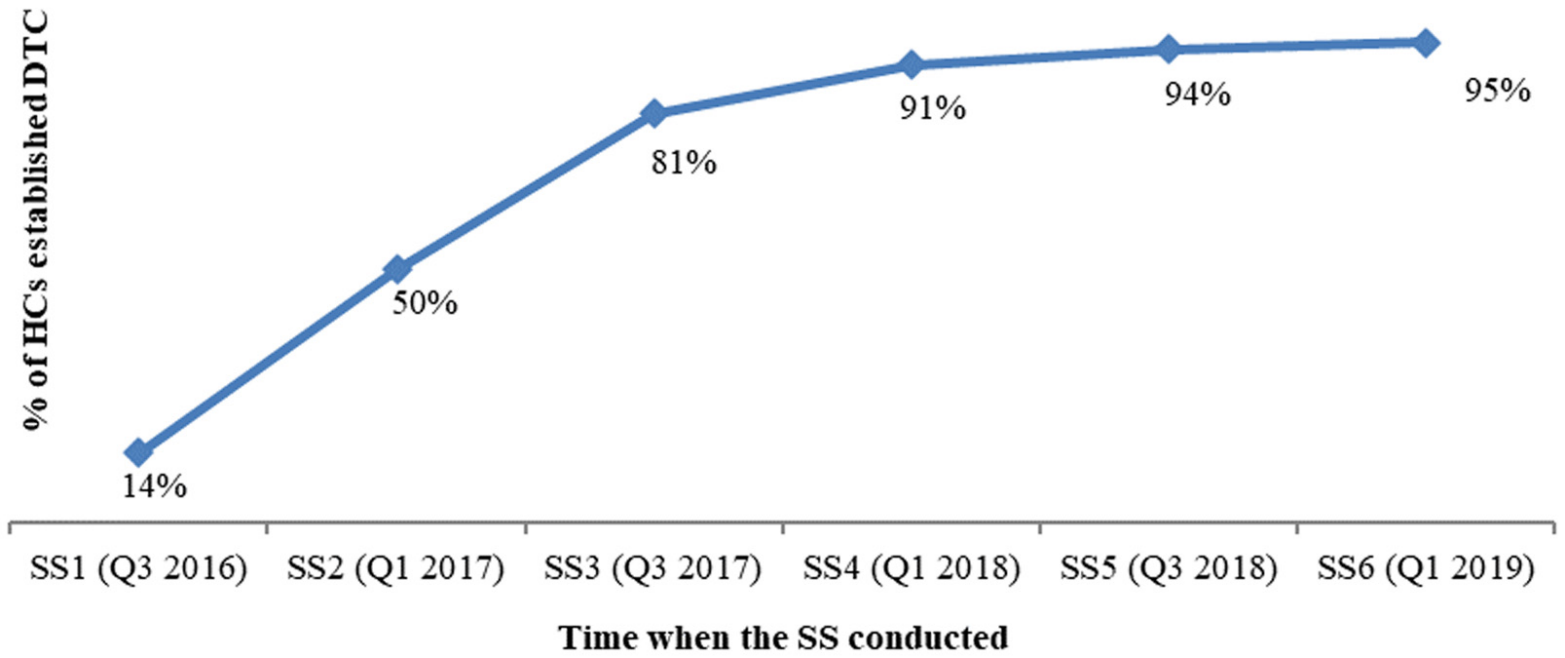
Trend analysis, % of HCs established DTC with official letter and DTC TOR. HF, health facility; DTC, Drug and Therapeutics Committee; TOR, terms of reference; HC, health center; SS, supportive supervision.

Apart from baseline and endline assessments, regular CHAI-Ethiopia/CSP SS data also revealed that DTC implementation has consistently improved from the first round SS to the sixth round SS as detailed in the following figures. DTC establishment improved from 83% to 100% of HFs in 2016 and 2019, respectively. Availability of DTC TOR increased from 20% to 99% among the HFs that established DTCs. Additional criterion considered during SS to confirm establishment of DTCs in the HFs is the availability of an official letter that has been dispatched by the HF to assign DTC members. From the HFs with an established DTC, the availability of an official letter improved from 51% (in 2016) to 96% (in 2019) ^13^. HFs that established a DTC with TOR and an official assignment letter improved from 14% to 95% in 2016 and 2019, respectively (see Figure 4).

**Figure 5.**
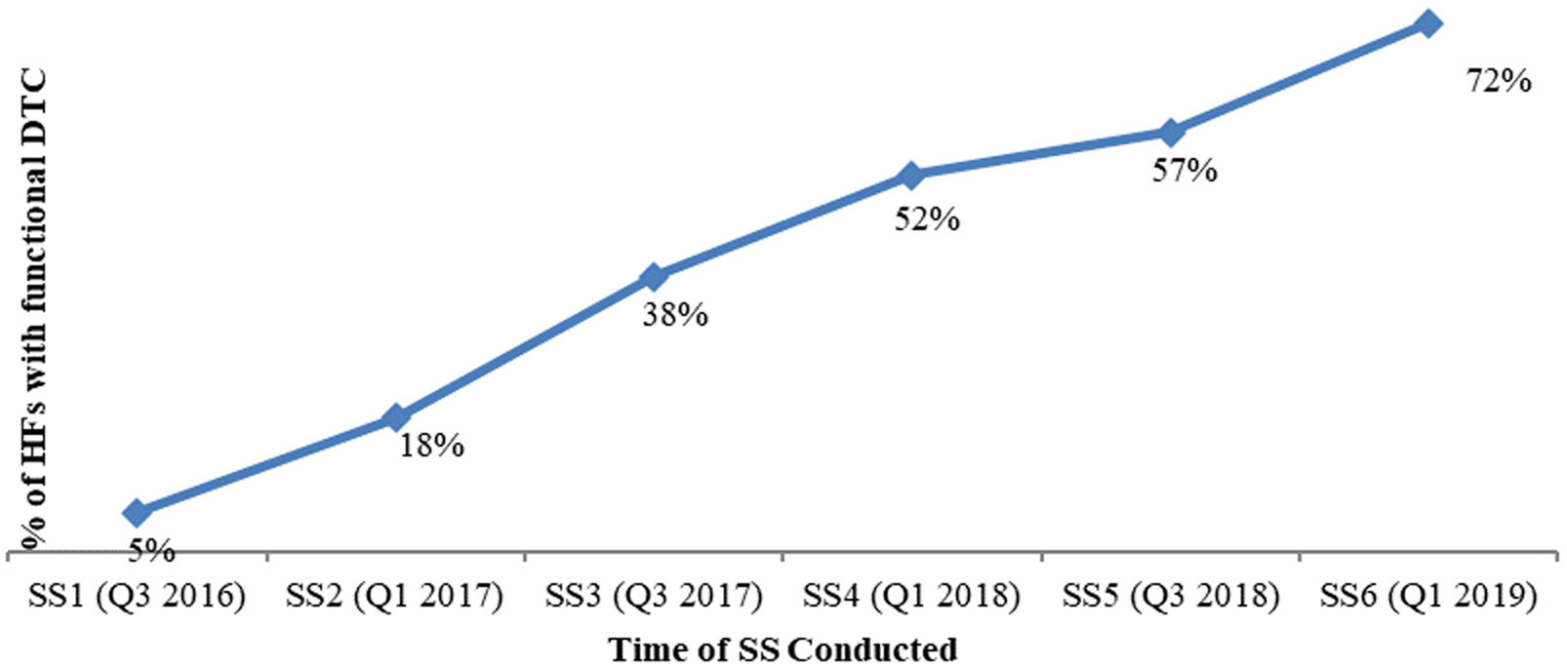
Trend analysis, % of HCs with DTC functional for all five indicators. HC, health center; DTC, Drug and Therapeutics Committee; SS, supporting supervision.

With regard to DTC functionality, in addition to the establishment criteria, three more criteria were assessed, namely (1) availability of the current year annual action plan for the DTC, (2) conducting regular DTC meetings with documented minutes and (3) availability of a facility specific medicine list. All progressively improved; availability of annual action plan improved from 25% to 90%, conducting regular meetings with minutes improved from 69% to 82% and availability of a HF specific medicine list improved from 47% to 95% HFs from 2016 to 2019, respectively. Overall, the percentage of HFs that fulfil the five DTC functionality criteria stated above improved from 5% (in 2016) to 72% as measured in 2019 ( Figure 5).

A chi-square test of independence was performed to examine the relationship between training of facility heads and pharmacy heads on DTCs and the availability of a functional DTC in the HF on the day of visit for the SS data. There is a significant association between the two variables (availability of trained person in the HF and functionality of DTC in the same HF) as shown in Table 4.

**Table 4.** Chi-square test for selected indicators of SS data.

Independent variable	Independent variable	Degree of freedom	Number of HFs (N)	chi-square value	p value
Head of this HF trained on DTC	Functional DTC is available in this HF *	7	2072	36.8	< .0001
Pharmacy head or head dispenser trained on DTC	Functional DTC is available in this HF *	7	2072	20.8	.004

* Note: Functional DTC is when satisfying all five indicators of DTC (members assigned through official letter, had DTC TOR, had action plan, conducted regular meetings and developed HF specific medicine list). *SS, supportive supervision; HF, health facility; DTC, Drug and Therapeutics Committee; TOR, terms of reference.*

Correspondingly, secondary data analysis of the endline assessment of the program revealed a similar association for the two variables (availability of trained person in the facility and functionality of DTC in the same facility) ( Table 5).

**Table 5.** Chi-square test for selected indicators of endline assessment data.

Independent variable	Independent variable	Degree of freedom	Number of HFs (N)	chi-square value	p value
Medical director or facility head trained on DTC	Functional DTC is available in this HF *	2	305	17.5	< .0001
Pharmacy head of the facility trained on DTC	Functional DTC is available in this HF *	1	305	25.5	< .0001

* Note: functional DTC is when satisfying all the thee indicators of DTC (had DTC TOR, conducted regular meetings and the meeting is documented). *DTC, Drug and Therapeutics Committee; HF, health facility; TOR, terms of reference.*

From the above two tests there is positive association where HFs that have a trained HF head and pharmacy head are highly likely to have functional a DTC.

Moreover, KII participants stressed that provision of blended DTC-IPLS SS skill training for woreda health office (WoHO) logistic officers was essential in providing technical support to their catchment HFs and standardization of reporting systems, because WoHO logistic officers are supervisors of their respective catchment HFs.

### Driving factors for DTC progress

Most of the KII participants forwarded the following points as key driving factors for DTC implementation improvements.

•   Government commitment to DTC inclusion in policy documents from EHRIG/EHCRIG to HSTP

•   RHB and majority of HFs commitment to integrating DTC activities in their annual planning

•   Intensive in-service training in recent years including TOT to RHBs, EPSA and ZHDs in order to establish TOT pools at regional level and cascade the training

•   Commencement of regular M&E like JSS using a standard checklist in collaboration with partners

•   Subsequent revision and sharpening of DTC training manuals and SOPs to make them user-friendly and contextualized

### Challenges of DTC implementation

Major challenges for DTC implementation that were forwarded by KII participants are summarized in the following thematic areas: policy and regulation related; human resource related M&E related.

***Policy and regulation related*.** Although DTCs are included in some strategic documents such as EHRIG/EHCRIG and HSTP, it is not well addressed in Ethiopian Health Policy/Drug Policy and thus is has limited legal framework for enforcement. There is no regulatory body to enforce DTC establishment and implementation of strengthening activities, effectively making these tasks voluntary rather than mandatory. There is also a lack of accountability/no reward or punishment mechanism clearly stated at a national level and applicable across the country. The committee members were not time favored to produce operational studies and to exercise their duties and responsibilities. Instability of the DTC mandate (the mandate has fallen to four different organization since DTC introduction in Ethiopia) is believed to be another bottleneck for its sustainable implementation or management ownership. Presently, the MOH is mandated to run DTCs, but the function is not well integrated into the routine health system from the national to facility level with respect to reporting and M&E systems. Weak networking and absence of strong structural support from MOH-RHBs-ZHDs and woreda health offices in strengthening DTCs have affected their implementation. There is also a lack of collaboration among the government stakeholders engaged in DTC work such as FMHACA/EFDA, EPSA, and the MOH. DTCs often have an overstretched scope of work and are asked to weigh in on many issues, which makes the DTC weak and lacking in the focus needed to accomplish core functionality criteria. There are several committees in a hospital and DTC committee members are also members of other committees, which share their time for DTC implementation. Furthermore, there is no DTC structure from the national to the last mile of the MOH administrative structure (woreda level) to support and guide facility-level DTCs. That is, the responsibility of DTCs is given to experts at each level as an additional task instead of being their main duty. Furthermore, no one is evaluating these experts for their role in the poor performance of a DTC. Poor commitment of individuals and/or HFs to implement DTCs according to the requirements is also a challenge; in facilities where there are committed leadership and individuals in the facility, DTC functionality is strong, but the reverse is observed in less committed facilities.

***Human resource related*.** There are shortages of pharmacy professionals to support DTC implementation in the facility. Staff turnover, particularly DTC trained staff turnover coupled with poor knowledge and skill transfer mechanisms greatly affects DTC performance in facilities, as does wrongly perceiving DTCs as only the role of pharmacists, when in reality they require a multi-disciplinary team and collective effort. Skill & knowledge gaps and lack of basic and gap filling training are also reasons for poor performance in some facilities. In addition, there is no short and long-term plan for regular or preservice training for all the professionals/members of DTCs (MDs, nurses, pharmacists, health officers, lab professionals) considering the curriculum revision.

***M&E related*.** There was no clear M&E system for DTCs. DTC indicators are not incorporated in HMIS/DHIS 2, the current MOH reporting platform. There is a lack of self-assessment at HFs of their DTC performance (there was/is no standardized key performance indicator for DTCs and DTC related activities were not included in the job description of DTC chairman, DTC secretary and members of the committees with a strong M&E framework). DTC focused ISS/JSS are neither regular nor well planned-with timely feedback and subsequent action points are not implemented across the country. Similarly, most review meetings did not include DTCs in their agenda of discussion. Poor documentation, starting from planning, performance monitoring and different reports/studies are common even among facilities that have functional DTCs. Of course, the recently developed MOH M&E framework tried to standardize DTC functionality criteria, but there are no well-established data capturing and follow-up mechanisms at the national level for key performance indicators.

### Limited engagement of private sectors

Although the private sector has a substantial role in the supply chain system and have an irreplaceable role in strengthening DTC their engagement in contributing the progress of DTC is very limited in the context of Ethiopia. DACA/FMHACA/EFDA have tried to push private hospitals to establish DTCs since 2008/2009, however, DTCs, have not yet started in private HFs.

## Discussions

This study presented a comprehensive overview of the DTC evolvement and its expanded scope in Ethiopia, and it included details of establishment and functionality criteria, driving factors, challenges, improvement measures, and recommendation actions. DTC establishment in health facilities (HFs) improved from 85% to 98% between 2015 and 2019 based on the baseline and end-line assessments carried out by CHAI/CSP. Although there is a marked difference in the establishment of DTCs between high and low-income countries, our finding is in line with studies conducted in high-income countries such as USA, UK, and Netherlands ^18, 19^. However, it is in contrast with the findings of studies conducted in Pakistan, Nigeria, Jordan, and Brazil which were 65%, 50%, 45%, and 11.6% respectively ^20– 23^.

Results of this study suggest that the main responsibilities of DTCs in Ethiopia are mainly rational drug use and pharmacy services like adverse drug reactions (ADR), antimicrobial resistance (AMR) containment, formulary list preparation, drug information system (DIS) establishment, Drug Use Evaluation (DUE), and ABC/VEN analysis. Worldwide, the main roles of the DTC include drug evaluation and the maintenance of hospitals' drug availability ^4, 24, 25^.

Conducting a regular meeting is one of the criteria for the functionality of DTC, in line with this our study found that the majority (82%) of the facilities conduct regular meetings which is slightly higher than a study conducted in UK (77%) ^26^. However, a study conducted in Jordan stated, it was only two-thirds of DTCs (66%) met and conducted regular meetings ^22, 23^.

The study found out that some of the challenges of DTC implementation were poor commitment of individuals and/or HFs to implement DTCs according to the requirements and lack of a regulatory body to enforce DTC establishment and implementation which makes the tasks of DTC voluntary rather than mandatory which is similar with a study conducted in Sierra Leone which found out that unmotivated DTC members and lack of recognition of DTC activities within the facilities ^26^. Moreover, a study conducted in Thailand found that poor performance monitoring and erratic national directives were some of the key gaps identified ^27^ which is similar to the findings of our study. Unlike the study conducted in Jordan which included private hospitals in its study ^22^, our study did not include any private hospitals in its study and which is considered one of the main limitations of this study. 

Based on the findings of the study, the following recommendation actions were forwarded: In order to improve DTC implementation, a national coordinating body for DTCs at the MOH or some type of advisory board should be established. Likewise, the same structure in RHBs, ZHDs & WoHO should be established and it should reflect its multidisciplinary approach as has been in practice at HF level. Moreover, a strong M&E system for DTCs should be in place and major DTC performance indicators should be included in all health programs and the national M&E system HMIS/DHS2. The recently developed DTC M&E framework should be implemented properly and should use standardized criteria for DTC functionality. Moreover, Strong documentation and information sharing within and between HFs on DTC best practices with standardized skill and knowledge transfer systems in HFs when there is staff turnover should be enhanced. 

Since there is a high turnover of trained staff and new entrants into the job, DTC training should continue (basic for new staff and refresher for previously trained staff) apart from mass orientation for all hospital staff. Similarly, training materials and job aids should be regularly updated and adequately printed, and distributed to all HFs and staff. 

The DTC’s major roles and/or functions and responsibilities should be incorporated into the preservice training/curriculum for all health professionals. On top of this, DTC should be included in the job description of the pharmacy head and facility head, as well as the person in charge of the DTC. It should be included in their performance evaluation and corrective actions should be taken accordingly.

Both the MOH and partners should give special attention to pastoralist areas to strengthen DTCs to improve overall SCM and pharmaceutical supplies, because to make DTC functional at facility level, concerted and coordinated efforts in terms of capacity building and follow-up mechanisms should be in place. Likewise, Public-Private Partnership (PPP) is also one of the key areas which didn’t get the attention of the MOH, therefore strengthening DTC establishment and functionality in private health facilities should be the focus of the MOH.

## Conclusion

As per the KII words, DTCs are a very important tool for practically addressing the overall challenges of health programs of a given HF as well as an important tool for health care improvement. In HFs where the DTC is consistently functional, wastage rates decreased and in return availability of essential drugs improved. Similarly, knowledge of prescribers, dispensers and patients improved. Quantification of medicines, medical supplies and medical equipment also improved in that almost all HFs sent their approved quantification via the DTC. Some reporting and requesting form (RRF) have been evaluated by DTC members, which results in improvement of RRF data quality; most HFs use standard prescriptions, improving counselling and dispensing times; and prescribing patterns have improved due to the assessments of prescribing indicators. Provision of blended DTC-IPLS SS skill training for WoHO logistic officers has been found to be essential in providing technical support to their catchment HFs and standardization of reporting systems.

As per the investigators’ summary, the DTC is an old multidisciplinary platform in Ethiopia. It was introduced to Ethiopia in the early 1980’s. Throughout this time, DTC implementation was mandated to different institutions. Currently, the mandate is with the MOH. Although DTCs were started 30 years ago in Ethiopia, their implementation has been lagging for various reasons, mainly due to frequent changes in DTC mandate and absence of clear roles and responsibilities for actors at all levels. Recently, the MOH as well as partners have given due attention to the implementation of DTCs so that it is included in national documents such as HSTP, EHRIG and EHCRIG. Training materials have been improved and SOPs developed over time. Several thousand health professionals have been trained on DTCs. Although it is persistently affected by high turnover, currently, availability of both trained facility heads and pharmacy heads of HFs on DTC at the time of visit has increased from 6% to 43% from baseline (2015) to endline (2019) assessments, respectively, in CHAI-supported HFs in Amhara, Oromia, SNNP and Tigray regions. Similarly, in those regions, establishment of DTCs in HFs reached nearly 100%, although functionality of DTCs is still about 63%. Moreover, the above achievements were not properly studied in pastoralist settings, in which the establishment and functionality of DTCs at HFs was not properly known. The assessment also revealed that a strong positive association was observed between the availability of DTC trained facility management persons and functionality of DTCs. That means the more closely HFs are supported, the greater the functionality of the DTC.

## Data Availability

Dryad: Drug and Therapeutics Committee (DTC) evolvement and expanded scope in Ethiopia. https://doi.org/10.5061/dryad.gqnk98smd ^10^. This project contains the following underlying data: DTC_study_data_from_CHAI_Ethiopia_CSP_Endline_Assessment_(HC)_de-identifies.csv DTC_Supportive_Supervision_CHAI_Ethiopia_(HC)_Data_(de-identified).csv DTC_Training_Database_CHAI_Ethiopia_De-identified.csv Key_infomant_interview_transcripts.zip (ZIP file containing transcripts in DOCX format) Dryad: Drug and Therapeutics Committee (DTC) evolvement and expanded scope in Ethiopia. https://doi.org/10.5061/dryad.gqnk98smd ^10^. This project contains the following extended data: DTC_Study_Supportive_Supervision_Checklist.docx Consent Note.docx (participant information sheet used to obtain participant consent) Data are available under the terms of the Creative Commons Zero "No rights reserved" data waiver (CC0 1.0 Public domain dedication).

## Footnotes

^1^ APTS: auditable pharmaceutical transactions and services, CBHI: community-based health insurance, RDF: revolving drug fund, AMR: Antimicrobial resistance, DIC: dug information center, DIS: drug information system.
