# Supplementary material for: Drug and Therapeutics Committee (DTC) evolvement and expanded scope in Ethiopia
Source: Gates Open Res. 2023 Mar 10;5:70. Originally published 2021 Mar 26. [Version 2] doi: 10.12688/gatesopenres.13200.2 (PMC10285046; doi:10.12688/gatesopenres.13200.2)
Supplement: Peer review history

# Peer review: Reviewer response for version 2

**DOI:** 10.21956/gatesopenres.15709.r32973

**Published:** 2023-06-13

**Authors:** Anteneh Girma Minas

**Affiliations:** 1 University of South Africa, Addis Ababa, Ethiopia

This is an article I reviewed a while ago. The comments provided that time are now addressed and endorsing the indexing of the article.

Is the work clearly and accurately presented and does it cite the current literature?

Yes

If applicable, is the statistical analysis and its interpretation appropriate?

Yes

Are all the source data underlying the results available to ensure full reproducibility?

Yes

Is the study design appropriate and is the work technically sound?

Yes

Are the conclusions drawn adequately supported by the results?

Yes

Are sufficient details of methods and analysis provided to allow replication by others?

Yes

Reviewer Expertise:

Public Health. Public Health Nutrition

I confirm that I have read this submission and believe that I have an appropriate level of expertise to confirm that it is of an acceptable scientific standard.

# Peer review: Reviewer response for version 2

**DOI:** 10.21956/gatesopenres.15709.r32972

**Published:** 2023-03-31

**Authors:** Joseph Fadare

**Affiliations:** 1 Department of Pharmacology and Therapeutics, College of Medicine, Ekiti State University, Ado-Ekiti, Nigeria

The authors have addressed the issues raised by the reviewer.

 Thank you.

Is the work clearly and accurately presented and does it cite the current literature?

No

If applicable, is the statistical analysis and its interpretation appropriate?

Yes

Are all the source data underlying the results available to ensure full reproducibility?

Yes

Is the study design appropriate and is the work technically sound?

Yes

Are the conclusions drawn adequately supported by the results?

Yes

Are sufficient details of methods and analysis provided to allow replication by others?

Yes

Reviewer Expertise:

Clinical Pharmacology and Therapeutics, Drug utilization research

I confirm that I have read this submission and believe that I have an appropriate level of expertise to confirm that it is of an acceptable scientific standard.

# Peer review: Reviewer response for version 1

**DOI:** 10.21956/gatesopenres.14408.r30535

**Published:** 2021-04-19

**Authors:** Anteneh Girma Minas

**Affiliations:** 1 University of South Africa, Addis Ababa, Ethiopia

I would like to congratulate the authors for their timely and appropriate study, which is/will be beneficial to contribute for the health systems strengthening in Ethiopia. I read the article with great interest and can command that it is well written with good flow of idea/language. Technically sound methods and analysis are followed, and the conclusion and recommendations also reflect the findings indicated in the study. However, I would like to provide the following comments/queries to the authors, and I hope it will help to further strengthen the quality of their study.

**ABSTRACT**

**Methods:** 'A descriptive study design was employed with mainly qualitative data collection methods and analysis. The assessment made use of both qualitative and quantitative data, generated from primary sources through key informant interviews and from secondary sources through desk review methods'.

 Comment:

- There seems a contradictory statement used in this paragraph. The first sentence indicated the use of qualitative methods, while the second indicate the use of both qualitative and quantitative methods. So, it is not clear which is which. Revision of this paragraph could make it clearer and less redundant use of words/phrases.

**Results: '**DTCs were introduced in Ethiopia in the early 1980’s. The mandate of DTCs has been given to four different government organizations since this time. As a result, its implementation was lagging'.

 Comments:

- I would replace the word ‘this’ with ‘that’…so it reads.’….to four different organizations since that time’. 
- There seems a missing element or disconnect between these two sentences, as (1) it is not if giving mandate to four different organizations was the reason for the lagging, (2) lack of coordination exists. (3) mandate was not clear.

**INTRODUCTION**

**Background: **Second paragraph, line #11: 'Currently, huge numbers of health facilities (HFs) both in developing and developed countries have established DTCs'.

 Comment:

- It is not clear what the word ‘currently’ implies. Mentioning of specific timeline/year could give a clearer picture of the status of DTC establishment.

**'Efforts, achievements and gaps in the establishment and strengthening of DTCs in Ethiopia'.**

 Comment:

- I would rephrase this sub title as as follow: ' **Efforts, achievements, and gaps in establishing and strengthening of DTCs in Ethiopia".**

Paragraph #1, line #11, 'Recently, the DTC guidelines and standard operating procedures (SOPs) were revised and tailored to a user-friendly and action-oriented approach. Using the revised SOP, the MOH and the Ethiopian Pharmaceuticals Supply Agency (EPSA) in collaboration with IPs have organized a number of cascading training sessions including training of trainers (TOT) for a huge number of health professionals (physicians, pharmacists, health officers and nurses) on DTCs'.

 Comment:

- I would avoid the word/adjective ‘huge’ from the sentence, as it reflects subjectivity and/or exaggerations.
- Additions/inclusion of a more recent literature, if available, would give a more recent status of DTC or related factors in Ethiopia. The most recent indicated in the document is the one from 2015. There could be some development and/or deterioration between 2015 and 2020/21. Or the authors could indicate lack of more recent publications/study after 2015, if there is none. At least the 2018 study by MOH could be mentioned/referenced.

**RATIONALE FOR THE STUDY**

 Comment:

- First paragraph, line #3, 'SNNPR' should be written in full, as it is mentioned the first time in the document.

**OBJECTIVES OF THE STUDY**

 General objective: 'The general objectives of this study are to review the evolution of DTCs in Ethiopia from their early years to current practice; identify the major hindering factors for their functionality and impact on the pharmaceutical SCM system and pharmacy service and propose feasible recommendations'.

 Comment:

- The general objectives reads as if the study assesses ‘hindering factors’ only, while the specific objectives include ‘enabling factors’, which are of course equally (or more) important as the hindering factors in informing the design and implementation of the pharmaceuticals SCM system in Ethiopia and other countries. I think it is worthwhile revising the general objective to ensure clearer harmony with the specific objectives.

**METHODS**

 SCOPE OF THE STUDY AND STUDY PERIOD

 'A desk review of available national and international documents from the introduction of DTCs into the country until the current situation was conducted'.

 Comment:

- The word current is misleading or not clear indicative. A reader reading this report after one or two years will perceive as if it is ‘current’ from the readers perspective even after a year. An indicative timeline of the review, example …. between the introduction of DTC in 1980s to 2020/201/ … would be more indicative.  

Study design

 'A descriptive study design was employed with mainly qualitative data collection methods and analyses'.

 Data type and sources

 'The assessment made use of both qualitative and quantitative data, generated from primary and secondary sources and collected through KIIs and desk review methods, respectively'.

 Comment:

- There seems some disconnect between the study design and the Data Source and type. The study design mentions use of qualitative data collection methods, while the data type and source indicate both qualitative and quantitative data. Wondering how quantitative data were collected through qualitative methods? I think the study design needs revision, as it appears from the document that both qualitative and quantitative methods were used. Results were quantified throughout the document. SPSS was also used.

**DESK REVIEWS**

 Comment

- What does ‘SS’ in the first paragraph, line #10 stand for?

**KEY INFORMANT INTERVIEW**

 'This qualitative study involved the collaborative efforts of a multidisciplinary research team coming from clinical background (medical doctors, internists, senior pharmacy professionals and senior public health professionals) with extensive experience in project evaluations and operational research'.

 Comment:

- I would review this sentence as follow: ‘This qualitative study involved the collaborative efforts of a multidisciplinary research team of medical doctors, internists, pharmacies and public health professionals with extensive experience in project evaluations and operational research.
- What is ‘IPs’ stands for? Line #13, paragraph 1 under KII

**RESULTS**

**Progress in scaling up DTC in Ethiopia**

***DTC establishment and functionality*** **.** 

 Comment:

 Paragraph #1:

- Line #4. “…. only 320 HFs were able to establish….’. What percentage do the 320 HFs represent? 
- Line #14 “…The national survey revealed that 97.8% of public hospitals had established a DTC’,... would be more informative if there is disaggregation of these data/findings by the regions included in the study.

Paragraph #2:

- Table 3: same as above, disaggregation of the findings on function by the regions under the study would be more informative to see/assess the progresses/variations by regions.

Driving factors for DTC progress

 Comment:

- Wondering why/how health facility leadership was not mentioned and indicated as a driving factor for DTC progress in Ethiopia, despite the study found/indicated significant statistical findings in the preceding sections.

Challenges of DTC implementation

 Comment:

- Wondering why the lack of private sector engagement not mentioned/indicated as a challenge for DTC progress in Ethiopia, regardless of a number of private health facilities in the four major regions covered in the study?

**RECOMMENDATIONS**

 Comment:

- Taking the absence of DTC establishment in private health facilities so far, recommendations related to private sector engagement and/or strengthen public private partnership in the DTC would be relevant for this study.

Is the work clearly and accurately presented and does it cite the current literature?

Yes

If applicable, is the statistical analysis and its interpretation appropriate?

Yes

Are all the source data underlying the results available to ensure full reproducibility?

Yes

Is the study design appropriate and is the work technically sound?

Yes

Are the conclusions drawn adequately supported by the results?

Yes

Are sufficient details of methods and analysis provided to allow replication by others?

Yes

Reviewer Expertise:

Public Health. Public Health Nutrition

I confirm that I have read this submission and believe that I have an appropriate level of expertise to confirm that it is of an acceptable scientific standard, however I have significant reservations, as outlined above.

# Peer review: Reviewer response for version 1

**DOI:** 10.21956/gatesopenres.14408.r30533

**Published:** 2021-04-12

**Authors:** Joseph Fadare

**Affiliations:** 1 Department of Pharmacology and Therapeutics, College of Medicine, Ekiti State University, Ado-Ekiti, Nigeria

This study documented the evolution of DTCs in Ethiopia over years including trends in functionality and potential impact on healthcare. Overall, it is well-conceived and written, however, there are some concerns:

1. The same data presented on Table 5 is repeated in Figure 4. There is a need to revise this.
2. The same data presented in Table 4 is repeated in Figure 3. This also needs to be revised.
3. There is no section on "discussion" of the results of this study. This is a major deficiency in this manuscript.
4. The section "recommendation" should be integrated into the discussion segment.
5. The references are mainly about studies or documents from Ethiopia. This is grossly inadequate. There is a need to broaden the scope of references to include similar research work from Africa and other parts of the world.

Is the work clearly and accurately presented and does it cite the current literature?

No

If applicable, is the statistical analysis and its interpretation appropriate?

Yes

Are all the source data underlying the results available to ensure full reproducibility?

Yes

Is the study design appropriate and is the work technically sound?

Yes

Are the conclusions drawn adequately supported by the results?

Yes

Are sufficient details of methods and analysis provided to allow replication by others?

Yes

Reviewer Expertise:

Clinical Pharmacology and Therapeutics, Drug utilization research

I confirm that I have read this submission and believe that I have an appropriate level of expertise to state that I do not consider it to be of an acceptable scientific standard, for reasons outlined above.
